# Inhibition of *Helicobacter pylori* and Its Associated Urease by Palmatine: Investigation on the Potential Mechanism

**DOI:** 10.1371/journal.pone.0168944

**Published:** 2017-01-03

**Authors:** Jiang-Tao Zhou, Cai-Lan Li, Li-Hua Tan, Yi-Fei Xu, Yu-Hong Liu, Zhi-Zhun Mo, Yao-Xing Dou, Rui Su, Zi-Ren Su, Ping Huang, Jian-Hui Xie

**Affiliations:** 1 Guangdong Provincial Key Laboratory of New Drug Development and Research of Chinese Medicine, School of Chinese Materia Medica, Guangzhou University of Chinese Medicine, Guangzhou, PR China; 2 The First Affiliated Hospital of Chinese Medicine, Guangzhou University of Chinese Medicine, Guangzhou, PR China; 3 Guangdong Provincial Key Laboratory of Clinical Research on Traditional Chinese Medicine Syndrome, The Second Affiliated Hospital, Guangzhou University of Chinese Medicine, Guangzhou, PR China; Universidad de Santiago de Compostela, SPAIN

## Abstract

In this paper, we evaluated the anti-*Helicobacter pylori* activity and the possible inhibitory effect on its associated urease by Palmatine (Pal) from *Coptis chinensis*, and explored the potential underlying mechanism. Results indicated that Pal exerted inhibitory effect on four tested *H*. *pylori* strains (ATCC 43504, NCTC 26695, SS1 and ICDC 111001) by the agar dilution test with minimum inhibitory concentration (MIC) values ranging from 100 to 200 μg/mL under neutral environment (pH 7.4), and from 75 to 100 μg/mL under acidic conditions (pH 5.3), respectively. Pal was observed to significantly inhibit both *H*. *pylori* urease (HPU) and jack bean urease (JBU) in a dose-dependent manner, with IC_50_ values of 0.53 ± 0.01 mM and 0.03 ± 0.00 mM, respectively, as compared with acetohydroxamic acid, a well-known urease inhibitor (0.07 ± 0.01 mM for HPU and 0.02 ± 0.00 mM for JBU, respectively). Kinetic analyses showed that the type of urease inhibition by Pal was noncompetitive for both HPU and JBU. Higher effectiveness of thiol protectors against urease inhibition than the competitive Ni^2+^ binding inhibitors was observed, indicating the essential role of the active-site sulfhydryl group in the urease inhibition by Pal. DTT reactivation assay indicated that the inhibition on the two ureases was reversible, further supporting that sulfhydryl group should be obligatory for urease inhibition by Pal. Furthermore, molecular docking study indicated that Pal interacted with the important sulfhydryl groups and inhibited the active enzymatic conformation through N-H **∙** π interaction, but did not interact with the active site Ni^2+^. Taken together, Pal was an effective inhibitor of *H*. *pylori* and its urease targeting the sulfhydryl groups, representing a promising candidate as novel urease inhibitor. This investigation also gave additional scientific support to the use of *C*. *chinensis* to treat *H*. *pylori*-related gastrointestinal diseases in traditional Chinese medicine. Pal might be a potentially beneficial therapy for gastritis and peptic ulcers induced by *H*. *pylori* infection and other urease-related diseases.

## Introduction

*Helicobacter pylori* is a Gram-negative spiral bacterium that colonizes the stomachs. *H*. *pylori* has been classified as the major risk factor of gastrointestinal diseases, including gastritis, gastric and duodenal ulceration and gastric carcinoma [1]. In the past decades, several treatment regimens were available to cure *H*. *pylori* infection. Among them, the most frequently used eradication regimen was the triple therapy consisting of amoxicillin, clarithromycin and proton-pump inhibitors [2]. Although this therapy has a success rate of 80%, the undesirable side effects, poor compliance, and antibiotic resistance cannot be ignored, which compromise its clinical application to some extent. Hence, there are continual efforts to discover potentially effective alternative options.

Urease (urea amidohydrolases, EC 3.5, 1.5) is known to be an important biological feature and major contributor to the pathologies induced by *H*. *pylori*. Urease initiates the hydrolysis of urea generating ammonia to neutralize stomach acid in order to create a suitable pH environment that the bacterium requires to survive and colonize [3]. In the structural unit of urease, nickel ions (Ni^2+^) and the sulfhydryl groups, at the active site of the enzyme, are essential for the catalytic effect of urease. The activity of urease has also been regarded as the influential factor of the nitrogen utilization in organisms. The urease causes the urea decomposition and re-absorption, which leads to abnormal environmental pH elevation and induces a variety of diseases in the human body [4]. Hence, urease is considered as a critical target in the research and exploitation of antibacterial agents [5]. Bioactive components of natural origin have been under enormous investigations as potential effective urease inhibitors for the treatment of *H*. *pylori* infection.

*Coptis chinensis*, known as ‘huanglian’ in Chinese, is traditionally used in China for the treatment of diarrhea and *H*. *pylori*-related gastrointestinal diseases [6]. Our earlier work has shown that the water extract of *C*. *chinensis* could inhibit HPU at a comparatively low concentration [7]. And the anti-urease effect of *C*. *chinensis* was more pronounced than its major active constituent berberine [8], implying that ingredients other than berberine might also play an important role in its urease inhibition. Palmatine (C_21_H_25_NO_4_, Pal), an active naturally occurring isoquinoline alkaloids, is another important bioactive component derived from *C*. *chinensis* besides berberine [9, 10]. Modern pharmacological investigations show that Pal exerts a broad variety of potentially useful pharmacological and therapeutic properties ranging from antibacterial to anticancer [11–18]. In China, Pal has been developed into an anti-inflammatory agent included in *Chinese Pharmacopoeia* (2015 Edition), and is widely used in clinical practice for the treatment of inflammatory diseases, including gynecological inflammation and digestive disorders like bacillary dysentery and enteritis, etc [19]. Previous report indicates that Pal and *C*. *chinensis* exhibited anti-*H*. *pylori* activity as well as inhibition on *H*. *pylori*-induced gastric inflammation [20]. However, little work has been done to illuminate the mechanism underlying their anti-*H*. *pylori* activity. Taking into account the crucial role that urease plays in the survival and gastric colonization of *H*. *pylori*, in this follow-up study, we endeavored to further investigate the possible inhibition of Pal on *H*. *pylori* and its associated urease, and probe the potential underlying mechanism.

## Materials and Methods

### Chemicals and reagents

**Fig 1:**
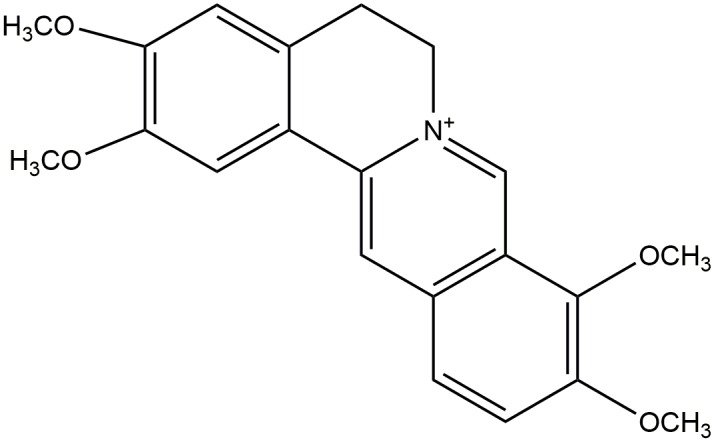
Chemical structure of Palmatine.

Palmatine (Pal, CAS number: 3486-67-7, the structure shown in Fig 1) was purchased from Sichuan Pure Chemical Industries (Sichuan, China). Metronidazole (Met, CAS number: 443-48-1,) was purchased from TOKU-E Company (Tokyo, Japan). Acetohydroxamic acid (AHA, CAS number: 546-88-3, purity: 98%), urea (Molecular Biology Reagent), jack bean urease (JBU, type III with specific activity 40.3 U/mg solid), HEPES (Amresco > 99%), L-cysteine (L-cys), glutathione (GSH), dithiothreithol (DTT), boric acid (BA) and sodium fluoride (NaF) were all purchased from Sigma-Aldrich (St Louis, MO, USA). Bradford Protein Assay Kit was purchased from Beyotime Institute of Biotechnology (Shanghai, China). HEPES buffer (20 mM, pH 7.5) was prepared by adjusting the pH with NaOH. Other chemicals and solvents were of analytical grade or HPLC grade and obtained from Guangzhou Chemical Reagent Factory (Guangzhou, China).

### The growth condition of *H*. *pylori* strains and determination of minimum inhibitory concentrations (MICs) by the agar dilution method

A total of four *H*. *pylori* strains were studied, including three reference strains ATCC 43504, NCTC 26695 and SS1 gifted from Professor Richard Ferrero, Monash University, Australia, and one clinical isolate ICDC 111001 obtained from Professor Zhang Jianzhong, Chinese Center of Disease Control and Prevention. All the strains were cultured on Columbia agar supplemented with bovine serum albumin for 72 h at 37°C under 98% humidity and microaerophilic conditions (5% O_2_, 10% CO_2_, and 85% N_2_).

The MICs of Pal against the four *H*. *pylori* strains were evaluated by using agar dilution method. Series of concentrations of 25, 50, 75, 100, 125, 150 and 200 μg/mL Pal were used. *H*. *pylori* strains were harvested and resuspended in PBS (1.0–2.0 McFarland were defined based on the strain properties). And 0.1 mL *H*. *pylori* suspension was inoculated and flooded in the Pal-containing or H_2_O (control) agar plate. After 72 h, the MIC values were determined as the lowest concentration at which there was no strain growth judged by visual examination. Metronidazole was used as the positive control. The experiments were evaluated under two different pH (7.4 and 5.3) conditions and repeated twice.

### Preparation of *H*. *pylori* urease and inhibition test

HPU was prepared as previously described [21]. The standard urease assay mixture included 150 mM urea in 20 mM HEPES buffer (pH 7.5). After addition of small aliquots of enzyme-containing solution of urease, the assay ran for 20 min. The enzymatic activity was evaluated spectrophotometrically by detecting the ammonia concentration at 595 nm according to the modified Berthelot (phenol-hypochlorite) method [22]. Urease activity was estimated with JBU as a standard, the specific activity of HPU was determined to be 17.0 U/mg. One unit of urease activity was defined as 1 μmol of ammonia released per min at 25°C. The amount of protein was determined by commercial Bradford Protein Assay Kit with BSA as a standard. The amount of ammonia released was decided from a standard curve. The activity of uninhibited urease was defined as the control activity of 100%.

Reaction mixtures comprising 0.25 mg/mL HPU or JBU and different concentrations of Pal or AHA at the volume ratio of 1:1, were incubated at 37°C in 20 mM HEPES buffer (pH 7.5) in the absence of urea. The reactions were initiated by mixing the urease and the inhibitor. The preincubation mixture was withdrawn at 20 min and immediately transferred into the standard assay mixtures for urease residual activity determination [23]. Inhibition rate (%) was calculated following the formula: I % = (1-activity with inhibitors/activity without inhibitors) × 100%. The IC_50_ was expressed as the concentration of inhibitor that decreased urease activity by 50% and calculated by plotting the percent of inhibition. The experiments were triply performed.

### Inhibition kinetic study

Michaelis constant *K*_*M*_ and the maximum velocity *v*_*max*_ values were evaluated by means of Lineweaver—Burk plots, using the initial velocities at different urea concentrations (ranging from 0.3125 to 15 mM), in the absence or presence of 1.00, 0.50, and 0.25 mM Pal. The concentrations of HPU and JBU were both 0.25 mg/mL in the assays. The values were obtained by applying nonlinear regression to the Michaelis-Menten equation. All experiments were performed in triplicate.

### Urease protection against Pal inactivation

For the protection with thiols-containing compounds, the mixture consisting of 0.25 mg/mL urease (HPU or JBU), 0.5 mM Pal (for both HPU and JBU) and 1.25 mM thiols (DTT, GSH and L-cys) was incubated for 5, 10, 20, and 40 min at 37°C, respectively. The control sample instead of the protectors contained a proper volume of respective buffer.

For the protection with inorganic compounds (BA and NaF), 0.25 mg/mL urease (HPU or JBU) was preincubated with 5 mM BA or 5 mM NaF for 20 min. Following preincubation, each sample of the protected urease was incubated with 0.5 mM Pal for 20 min. The samples containing a proper volume of respective buffer instead of Pal served as comparison with the abovementioned systems. The experiments were triply performed.

### Pal-thiol-urease interaction test

The reaction mixture consisted of 0.25 mg/mL HPU or JBU, 0.5 mM Pal (for both HPU and JBU), and 1.25 mM thiols (DTT, GSH, or L-cys) in 20 mM HEPES buffer (pH 7.5). The components of the incubation mixture were mixed according to three procedures: (1) Urease was added to the mixture after a 20-min co-incubation of Pal with thiol. (2) Pal was added to the mixture after a 20-min co-incubation of urease with thiol. (3) Thiols were added to the mixture after a 20-min co-incubation of urease with Pal. The incubation mixture containing all components was incubated for further 20 min and the activity of urease was determined. The experiments were triply performed.

### Reactivation of Pal-inactivated urease in the presence of DTT

The reactivation of Pal-inactivated urease was investigated by DTT application. The preincubation mixture consisted of 0.25 mg/mL HPU or JBU and 0.5 mM Pal in 20 mM HEPES buffer (pH 7.5). Then the mixture was further incubated with 1.25 mM DTT after 20 min. The enzymatic activity of urease was determined before and after the addition of DTT. After a while, aliquots were withdrawn and the urease residual activity was determined. The experiments were repeated three times.

### Molecular docking protocol

Docking studies were performed as our previous investigation [24]. Pal was docked onto the ligand binding sites of the two ureases by Auto-Dock version 4.0, graphical user interface AutoDock Tools (ADT1.5.2) and the hybrid Lamarckian Genetic Algorithm (LGA). The three-dimensional (3D) crystallographic structures of HPU (PDB ID-1E9Y) complexed with JBU (PDB 3LA4) were downloaded from the RCSB Protein Data Bank. The 3D structure (PDB format) of Pal was modeled by using chem 3D Ultra 8.0 software. For docking, we analyzed the 3D results using the PyMOL molecular graphics system [25]. The cubic grid box of 60 Å size (x, y, z) with a spacing of 0.5 Å and grid maps were created. The average coordinates of the two Ni^2+^ were in the center of the grid. Other docking parameters were set to the software’s default values. The docked complexes were geometry optimized and the results of the most favorable free energy of binding were chosen as the resultant complex structures. The distance (Å) between hydrogen bond forming residues was measured.

### Statistical analysis

GraphPad Prism 5 (GraphPad Software Inc.) software was used for statistics and plotting. Data were expressed as means ± standard deviation (SD). The differences of the results were assessed using a one-way analysis of variance (ANOVA) followed by Dunnett's test. The analysis was performed using SPSS 13.0 software (SPSS Inc.). A significant difference was identified at *P* < 0.05.

## Result

### Antibacterial activity

The anti-*H*. *pylori* activity of Pal in the MIC assay was shown in Table 1. The MIC values of Pal against the four *H*. *pylori* strains under neutral conditions (pH 7.4) were from 100 to 200 μg/mL, and the MICs under acidic environment (pH 5.3) ranged from 75 to 100 μg/mL. The result indicated that Pal exhibited inhibitory activity against *H*. *pylori* under both neutral and acidic conditions, and the inhibitory effect seemed to be more obvious in acidic condition than the neutral environment. Metronidazole, a well-known antibiotic, was used as a positive control. The MIC values of metronidazole were summarized in Table 1.

**Table 1:** Minimal inhibitory concentrations (MICs) of Pal and Met.

*H*. *pylori* strains	MICs (μg/mL)^a^ (pH 7.4)		MICs (μg/mL) (pH 5.3)	
	Pal	Met	Pal	Met
ATCC 43504	100	0.5	75	0.5
NCTC 26695	200	2.0	100	2.0
SS1	100	0.5	75	0.5
ICDC 111001	100	2.0	75	2.0

^a^ Data represent MIC values observed from two independent experiments. Pal, palmatine. Met, metronidazole.

### Inhibitory effects of Pal on urease

**Fig 2:**
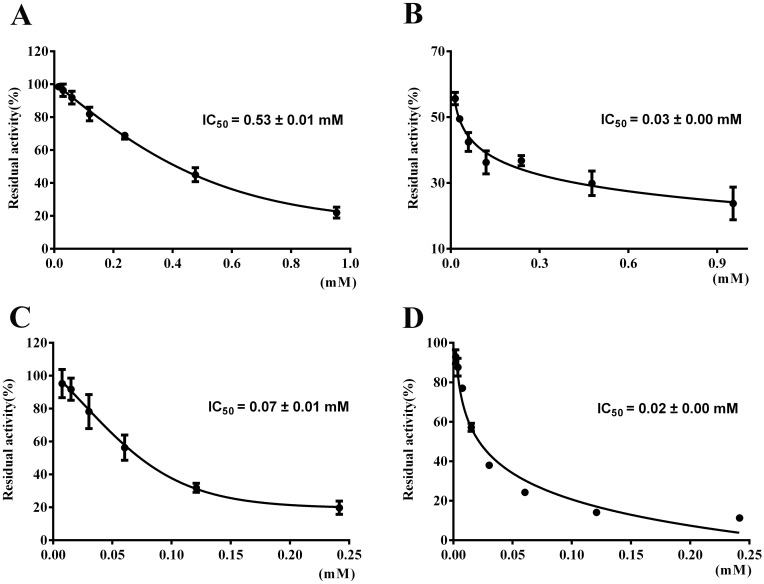
Effect of Pal on the activities of HPU and JBU. HPU was treated with Pal (**A**) and AHA (**C**); JBU was incubated with Pal (**B**) and AHA (**D**). The residual activity was calculated as percent of the control activity. The results summarized here are the mean values ± SD from three independent experiments.

The inhibition progress curves as a function of urease activity versus the incubation concentration of Pal with the two ureases were illustrated in Fig 2. It was observed that Pal inhibited the urease activity with IC_50_ values of 0.53 ± 0.01 mM and 0.03 ± 0.00 mM toward HPU and JBU, respectively. The IC_50_ values of AHA, a well-known urease inactivator employed as a positive drug, were 0.07 ± 0.01 mM and 0.02 ± 0.00 mM for HPU and JBU, respectively. The result revealed that Pal markedly inhibited the enzymatic activities of both ureases, and the IC_50_ value of Pal for JBU was similar to that of AHA.

### Determination of *K*_*M*_ and *v*_*max*_

**Fig 3:**
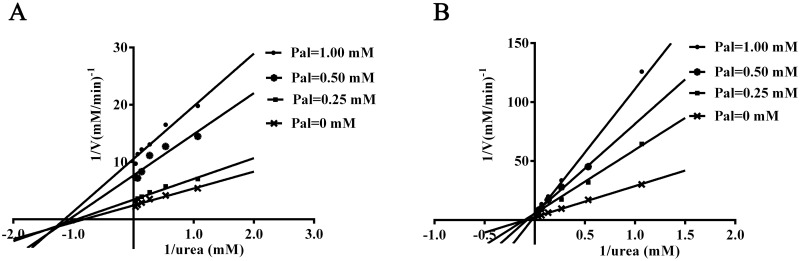
Lineweaver-Burk plots of HPU (A) and JBU (B) inhibition by Pal versus reciprocal of substrate concentrations (0.3125–15 mM) in the absence or presence of 1.00, 0.50, and 0.25 mM Pal. The concentrations of both ureases were 0.25 mg/mL. Data were expressed as means ± SD from three independent experiments.

In the present study, the enzymatic kinetics were determined by applying nonlinear regression to the Michaelis-Menten equation, in the presence or absence of various concentrations of Pal. The inhibition pattern was determined by the analysis of Lineweaver-Burk plots for elucidating the mechanism of inhibition. Kinetic analysis revealed that HPU followed a Michaelis—Menten kinetics in the presence of Pal. As shown in Fig 3A and 3B, the *K*_*M*_ and *v*_*max*_ of the ureolytic reaction were 1.20 ± 0.07 mM and 0.40 ±0.02 mM/min for HPU, and 8.30 ± 1.14 mM and 0.36 ± 0.02 mM/min for JBU, respectively. Moreover, the *K*_*M*_ value did not vary significantly, while the *v*_*max*_ value decreased as the Pal concentration increased, indicating Pal was a noncompetitive inhibitor of both HPU and JBU.

### Urease protection against Pal inactivation

**Fig 4:**
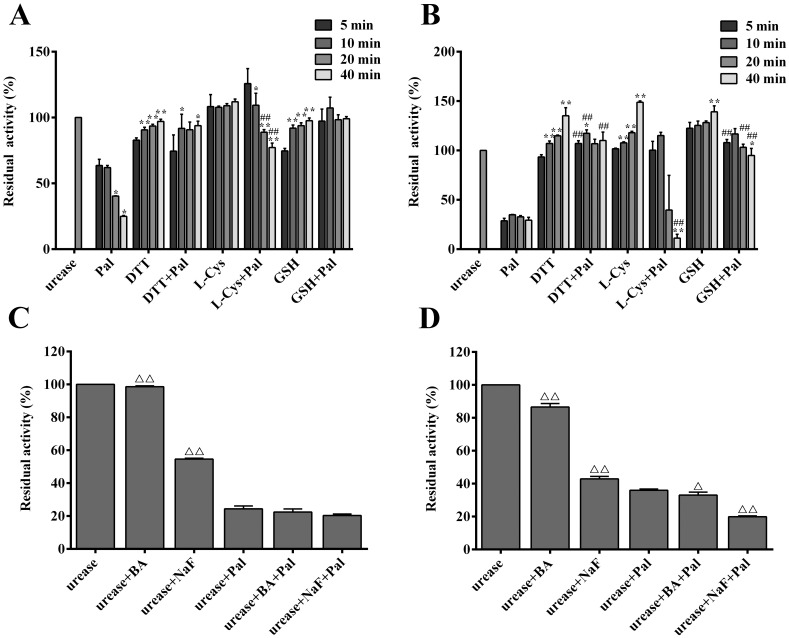
Effect of thiol-containing compounds and inorganic compounds on the inhibition of urease activity by Pal. Thiol reagents protected against HPU (**A**) and JBU (**B**) inactivation by Pal. Inorganic compounds protected against HPU (**C**) and JBU (**D**) inhibition by Pal. The concentrations of both ureases were 0.25 mg/mL. Concentrations of all thiol-containing compounds were 1.25 mM and the competitive inhibitors were 5 mM. The concentration of Pal was 0.5 mM for both HPU and JBU in the assays. After 5, 10, 20, and 40 min of incubation, residual urease activity was assayed respectively. Residual activity values were expressed as percent of the control activity. The residual activity of urease in the presence of Pal without the protector or urease in the presence of protector without the Pal was given for comparison. Results presented here are the mean value of n = 3 ± SD. *P < 0.05 and **P < 0.01 were considered as significant differences when compared with control (the first column of each group), ^##^*P* < 0.01 in comparison to the control (urease in the presence of protector without the Pal group), and ^Δ^*P* < 0.05 and ^ΔΔ^*P* < 0.01 in comparison to the control (urease in the presence of Pal without the protector), as determined by one-way ANOVA.

In the current research, the role of thiol protectors (DTT, GSH, and L‑cys) in urease inactivation by Pal was investigated by comparing the thiol-free system at four time points of incubation (5, 10, 20, and 40 min). According to Fig 4A and 4B, it was found that the inhibitory effects of Pal against HPU and JBU were time-dependent, and the thiol-containing compounds could significantly alleviate urease from loss of enzymatic activity by Pal, indicating Pal might interact with the sulfhydryl groups of urease. Moreover, the urease protection against inactivation by Pal was not obviously dependent on the incubation time.

To investigate whether the active site nickel ions was involved in the inactivation by Pal, two active-site competitive binding inhibitors (NaF and BA) were also employed in the urease protection experiment. As shown in Fig 4C and 4D, in the presence of BA or NaF, the activity of enzyme inactivated by Pal decreased from 98.6%, 54.6% to 22.42%, 20.35% and 86.6%, 42.9% to 33.08%, 19.91% toward HPU and JBU, respectively, even lower than that in the presence of Pal and NaF or BA alone. The data suggested that there was probably a synergic relationship between Pal and NaF or BA. Taken together, higher effectiveness of thiol protectors against Pal inhibition than the inorganic compounds suggested that the urease inhibition might be attributed to the interaction of Pal with the active-site sulfhydryl group.

### Pal-thiol-urease interaction test

**Fig 5:**
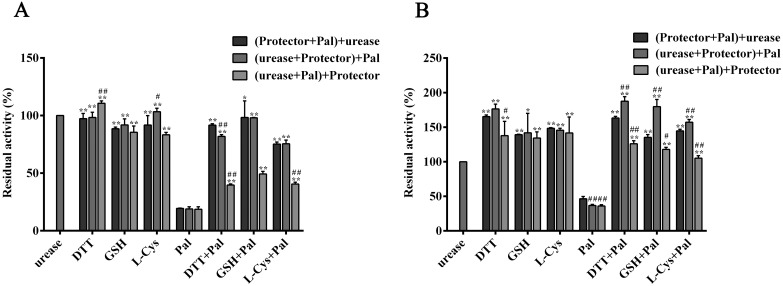
Effect of different component preincubation orders on HPU (A) and JBU (B) inactivated by Pal. The initial 20 min preincubation mixture contained components given in brackets. Further preincubation was continued for another 20 min after the addition of the last component (given outside the brackets). The concentrations of both ureases were 0.25 mg/mL. The concentration of all thiols used was 1.25 mM and Pal was 0.5 mM. Residual activity values were expressed as percent of the control activity. Results summarized here are the mean value of n = 3 ± SD. The residual activity of urease in the presence of Pal without the thiols is given for comparison. *P < 0.05 and **P < 0.01 were considered as significant differences when compared with control value (urease in the presence of Pal without the protector), and ^#^*P* < 0.05 and ^##^*P* < 0.01 in comparison to the control (the first column of each group) and, as determined by one-way ANOVA.

As depicted in Fig 5, all thiols were observed to afford protection from the loss of activities of the two ureases against Pal in different addition orders (the initial preincubation mixture contained ingredients given in brackets, and further preincubation was performed after the addition of the last component given outside the brackets), when compared to the control. As compared to HPU, the protection of thiols was more obvious for JBU. Furthermore, the protection of thiols was significantly associated with the addition order of urease, inhibitors, and the protectors. The mixture with initial preincubation of protectors evidently afforded more pronounced protective effect than the final addition of protectors concerning the effect of preincubation order.

### Reactivation of Pal-inactivated urease by DTT

**Fig 6:**
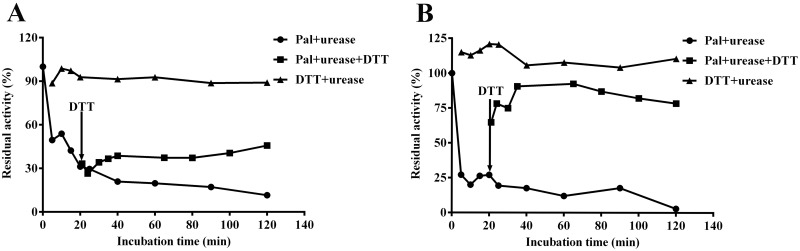
Reversal of Pal-inhibited HPU (A) and JBU (B) activity by DTT. The urease enzymatic activity was inhibited by Pal (•), and DTT (▪) was added post 20 min. In the assay system, 1.25 mM DTT and 0.5 mM Pal were used for both ureases (0.25 mg/mL). Activity of Pal-inhibited urease was monitored before and after DTT addition, 1.25 mM DTT without was used as a control. Results summarized here are the mean value of n = 3 ± SD.

In this section, DTT was employed to examine the stability of the urease-inhibitor complex. 1.25 mM DTT without Pal was used as a control. By adding DTT after 20 min co-incubation of Pal with JBU or HPU, the urease activity was observed to recover time-dependently. Compared with the urease activity of addition of 1.25 mM DTT without Pal (The HPU or JBU activity was ca. 100%), the HPU activity was partly restored to ca. 46% (Fig 6A), and JBU restored to ca. 78% (Fig 6B) of their initial activity. However, the HPU and JBU activities were restored compared with their initial activity (Before adding DTT after 20 min co-incubation of JBU or HPU with Pal), which did not cause any further inactivation after DTT addition. Restoration of the activity of Pal-modified urease with DTT was congruent with the result of urease protection experiment by thiol-containing compounds abovementioned, further supporting that the inactivation of urease might involve interaction of Pal to the sulfhydryl groups at the active site of ureases.

### Molecular docking study

**Fig 7:**
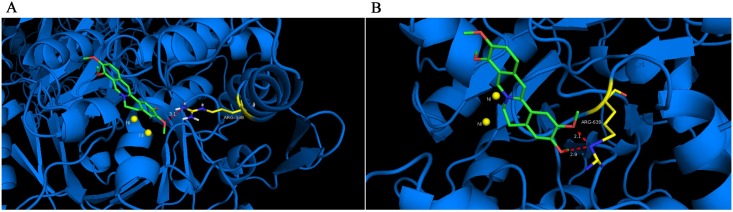
Docked conformation of of Pal in the catalytic site of HPU (A) and JBU (B). Hydrogen bonding interactions are shown by dashes. These figures were created by PyMol. (colored by atom: carbon is green, nitrogen is blue, oxygen is red, hydrogen is gray and sulfur is yellow)

**Fig 8:**
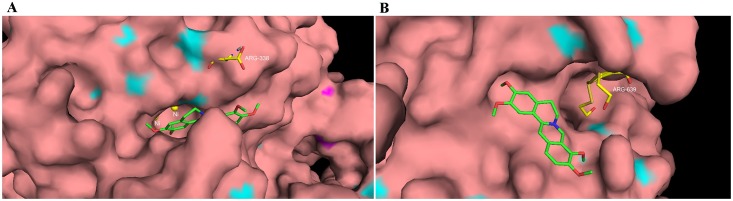
Surface representation of the active-site flap of urease with Pal, shown at the entrance of the binding pocket. **A: HPU, B: JPU.** (colored by atom: carbon is green, nitrogen is blue, oxygen is red, hydrogen is gray and sulfur is yellow).

To give an explanation and understanding of the observed appreciable activity and mechanism revealed by the protective experiment, molecular docking of Pal into the binding site of of HPU and JBU was performed based on the HPU (1E9Y) and JBU (3LA4) complex structures, and the best possible binding modes of Pal were depicted as cartoon model (Fig 7) and enzyme surface (Fig 8), respectively. From the docking conformation, it was observed that Pal was well filled in the active pocket of the two ureases, tightly anchoring the helix-turn-helix motif over the active-site cavity through O-H∙ ∙ ∙N hydrogen bonding interactions for both HPU and JBU, which prevented the flap of the urease active-site cavity from backing to the close position. For HPU, 2-OCH_3_ of Pal as the hydrogen bond donor, was found between the OH and the backbone N atom of ARG-338 (H∙ ∙ ∙N distance = 3.1 Å), which was located on the mobile flap closing the active site of the enzyme. And for JBU, 2-OCH_3_ and 3-OCH_3_ of Pal formed two strong O-H∙ ∙ ∙N hydrogen bond (H∙ ∙ ∙N distance = 2.1 Å, H∙ ∙ ∙N distance = 2.9 Å) to the backbone N atom of ARG-639. These observations were soundly supportive of the involvement of the active-site sulfhydryl groups in the inhibition process, as substantiated in the protective experiment performed with the active-site binding inhibitors and reactivation assay.

## Discussion

*Coptis chinensis* has been commonly employed in traditional oriental medicine for the treatment of various diseases, including *H*. *pylori*-related gastrointestinal disorders. Pal was one of the major bioactive protoberberine-type alkaloids of *Coptis chinensis*. Han et al. previously reported that Pal exhibited inhibitory effect against *H*. *pylori* (KCCM 41351) [6]. Jung et al. reported that Pal from *C*. *chinensis* had significantly inhibitory activities against *H*. *pylori* and gastric damage, of which the *in vivo* anti-*H*. *pylori* effect of Pal was similar to that of the positive control ampicillin [20]. In the present study, Pal was observed to exhibit suppressive effect towards four *H*. *pylori* strains including three reference strains (ATCC 43504, NCTC 26695, SS1) and one clinical isolate (ICDC 111001) under neutral and acidic conditions. And the anti-*H*. *pylori* activity of Pal was superior under acidic environment as compared with that of the neutral condition. This finding suggested that Pal might exert inhibitory effect toward *H*. *pylori* in the stomach, which was in congruent with the the *in vivo* anti- *H*. *pylori* effect in the previous report [20].

Urease has been implicated as an essential colonization and virulent factor of *H*. *pylori*. Urease allows *H*. *pylori* to survive in the low pH environment of the stomach during colonization by the hydrolysis of urea into ammonia and carbon dioxide to neutralize the bacterial microenvironment [26, 27]. Therefore, substances that could decrease the urease activity of *H*. *pylori* would be a potential anti-*H*. *pylori* agent. And exploration of safe and efficacious urease inhibitors derived from medicinal herbs is gaining increasing importance as an alternate therapy against *H*. *pylori* infections.

Urease of different origins such as plants and bacteria composes of inconsistent types of subunits, nevertheless, ureases share similar amino acid sequences and thus exhibit common catalytic characteristics. Urease from jack bean (*Canavalia ensiformis*, JBU), the first enzyme to be crystallized and the best-characterized urease [28], is deemed as a model urease for the inhibitory mechanism research. In this study, JBU was employed for comparison and reference purpose. In order to probe the mechanism of inhibition of Pal on *H*. *pylori*, Pal was evaluated for its anti-urease capacity. From the results obtained, it was found that Pal could effectively inhibit the activities of both HPU and JBU. This result indicated that the urease-inhibitory activity of Pal might be closely associated with the suppression of *H*. *pylori* growth.

The most pivotal catalytic characteristics of ureases are the cysteinyl residues bearing thiol groups and Ni^2+^ present in the active site of enzymes, crucially responsible for the enzymatic activity. So far, there are two well-defined urease protective agents frequently employed to probe the inhibition mechanism. One is the thiol-containing reagents including dithiol (DTT) and monothiols (e.g. L-cys and GSH), and the other is the inorganic compounds, such as BA (a classical competitive urease inhibitor) and NaF (a competitive slow binding urease inhibitor), which were reported to inhibit the urease by interacting with Ni^2+^ in the urease active site. In equilibration with the urease, both types of protectors occupy the active site of urease to restrain the accessibility of inactivators to the active-site of urease. Specifically, the thiol reagents inhibit the urease via interaction with the active-site sulfhydryl group of urease, while inorganics have been defined as competitive inhibitors, binding to its active site Ni^2+^ resulting in urease inhibition [29, 30]. In the present study, the thiol-containing compounds were found to exhibit a more appreciable protective effect when compared with the inorganic compounds. The thiol protectors (DTT, GSH and L-cys) evidently slowed down the rate of inactivation, while the unconspicuous protection by inorganic compounds from the inactivation was observed, suggesting that the target of inhibition of urease by Pal was possibly the active-site sulfhydryl groups. Since the inhibitory effect of NaF or BA on HPU and JBU displayed a possible synergic mode between Pal and NaF or BA, it was further proved that the active site sulfhydryl group was the potential target responsible for urease inhibition process.

Enzyme kinetic study is pivotal for the determination of the inhibition mechanism and sites of inhibitory binding. The Lineweaver-Burk plot was used to study the inhibition type in enzyme kinetics. According to the variations of *K*_*M*_ and *v*_*max*_ from the Lineweaver-Burk plots, it was proposed that the kinetic behavior might be noncompetitive for both HPU and JBU, implying Pal and substrate both got access to the urease non-competitively. In addition, the anti-urease activity of Pal was significantly affected by the incubation time. However, the thiol protection against urease inactivation by Pal was not relevant to the incubation time, but closely related to the addition order of the incubators. The results showed that the thiol-protectors might quickly combine with the sulfhydryl groups and restrict the accessibility of the inhibitor. In order to further prove whether the inactivation was reversible, DTT was employed in the reactivation of Pal-inactivated urease assay. More obvious protective effect exerted by DTT was observed in JBU as compared with that of HPU. Particularly, nearly 80% of JBU activity was recovered, but only 46% of total enzymatic activity of HPU was suppressed by Pal. This might be due to the specificity in the mode of interaction between HPU and JBU [31].

Furthermore, it was also supported by molecular docking simulation. As the results depicted, Pal was found to make hydrogen bonding interactions with ARG-338 (HPU) and ARG-639 (JBU), which were located on the urease active-site mobile flap. However, the interaction with the active-site Ni^2+^ was not found to be obvious. It was shown that 2-OCH_3_ group affected the molecular conformation interacting with HPU, which enhanced the binding with ARG-338, a key residue reported previously as catalytic residue in the active site [32], resulting in lowered enzymatic activity. As far as JBU was concerned, 2-OCH_3_ and 3-OCH_3_ groups were observed to interact with ARG-639, a key residue located at the mobile flap covering the active site, hence preserving the flap in the open conformation and leading to urease inactivation. Results from the molecular docking simulation further supported our earlier conclusion from the urease protection experiments and DTT re-activation assay, which suggested the pivotal participation of the active-site sulfhydryl group in the inactivation of urease by Pal.

Hence, our data indicated that the interaction with the sulfhydryl group of the active site of urease was closely associated with the anti-urease activity of Pal, which might be one of the mechanisms by which Pal inhibited the growth of *H*. *pylori*. Pal derived from *C*. *chinensis* might play an important role in the protection and therapy of *H*. *pylori*-related gastrointestinal disease. However, the more detailed underlying mechanism merited further investigation.

In China, Pal has been developed as a safe and effective anti-inflammatory agent in clinic for the treatment of gynecological inflammation, digestive inflammatory disorders and urinary infection, etc. Previous study indicated that the anti-ulcerogenic activity of Pal was better than that of the *C*. *chinensis* extract and its active principle berberine, and the *in vivo* anti-*H*. *pylori* activity of Pal and berberine was similar to that of ampicillin [20]. It was also found that the toxicity of Pal was the minimal among the four major *C*. *chinensis* alkaloids (berberine, coptisine, palmatine and epiberberine), while berberine was relatively more toxic in both *in vitro* and *in vivo* assays [33]. In our previous investigation, the IC_50_ of berberine against HPU and JBU was 10.20 ± 1.10 mM and above 13.00 mM, respectively [8], indicating the anti-urease activity of berberine was weaker as compared with that of Pal. Hence, it was concluded that Pal might exert more potent gastroprotective, anti-*H*. *pylori* and urease-inhibitory effect than berberine, and had a more favorable safety profile.

The current first-line monotherapeutic agent for *H*. *pylori* treatment either focuses on the direct anti-*H*. *pylori* effect or gastroprotective property, and none of them harbor simultaneous anti-*H*. *pylori*, HPU-inhibitory and gastroprotective activities. Based on its simultaneous anti-*H*. *pylori*, HPU-inhibitory and gastroprotective properties, Pal was believed to possess good potential for further development into a safe and efficacious non-antibiotic therapeutic agent in the management of *H*. *pylori*-related gastrointestinal disorders, and the discovery of Pal as a safe and efficacious urease inhibitor and rational characterization of its underlying mechanisms will undoubtedly add new therapeutic dimensions to its current clinical application and also provide a foundation and justification for further research as a potential complementary therapeutic agent to the current conventional medications.

## Conclusions

It was concluded that palmatine might suppress *H*. *pylori* growth, at least in part, through inhibition of its urease. The concentration-dependent, reversible, and noncompetitive inhibition against urease by palmatine might be attributed to its interaction with the sulfhydryl group of the active site of urease. Our work gave additional scientific support to the use of *C*. *chinensis* to treat *H*. *pylori*-related gastrointestinal disorders and might extend the clinical indications of palmatine. Palmatine deserves to be further exploited as a promising naturally-occuring urease inhibitor for the treatment of *H*. *pylori* infection and other urease-related diseases.

## Supporting Information

S1 File Effects of Pal and Met on the growth of four *H*. *pylori* strains (ATCC 43504, NCTC 26695, SS1 and ICDC 111001) under neutral (pH 7.4) and acidic (pH 5.3) conditions by the agar dilution method. All the strains were cultured on Columbia agar supplemented with bovine serum albumin for 72 h at 37°C under 98% humidity and microaerophilic conditions (5% O_2_, 10% CO_2_, and 85% N_2_). And 0.1 mL *H*. *pylori* suspension was inoculated and flooded in the Pal or Met-containing or H_2_O (control) agar plate. After 72 h, the effects of Pal and Met on the growth of *H*. *pylori* were determined.
(DOCX)

S2 File The raw data of Figs 2, 3, 4, 5 and 6. (XLSX)
