# Supplementary material for: Inhibition of Helicobacter pylori and Its Associated Urease by Palmatine: Investigation on the Potential Mechanism
Source: PLoS One. 2017 Jan 3;12(1):e0168944. doi: 10.1371/journal.pone.0168944 (PMC5207512; doi:10.1371/journal.pone.0168944)
Supplement: S1 File — All the strains were cultured on Columbia agar supplemented with bovine serum albumin for 72 h at 37°C under 98% humidity and microaerophilic conditions (5% O2, 10% CO2, and 85% N2). And 0.1 mL H. pylori suspension was inoculated and flooded in the Pal or Met-containing or H2O (control) agar plate. After 72 h, the effects of Pal and Met on the growth of H. pylori were determined. (DOCX) [file pone.0168944.s001.docx]

**Fig S1 Effects of Pal and Met on the growth of *H. pylori* ATCC 43504 under neutral condition (pH 7.4) at different concentrations by the agar dilution method.** After 72 h, the effects of Pal and Met on the growth of *H. pylori* were determined. **A**. Control group for Pal; **B**. Pal (75 µg/mL); **C**. Pal (100 µg/mL); **D**. Pal (125 µg/mL); **E**. Control group for Met; **F**. Met (0.25 µg/mL); **G**. Met (0.5 µg/mL); **H**. Met (1 µg/mL).


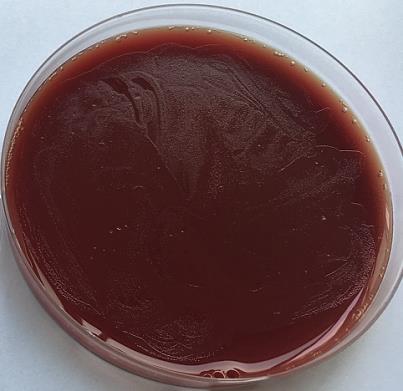

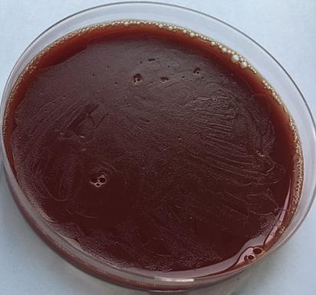


**A**


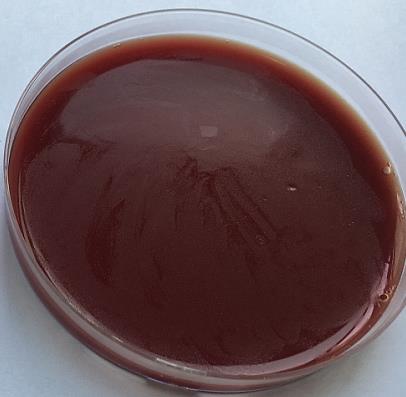

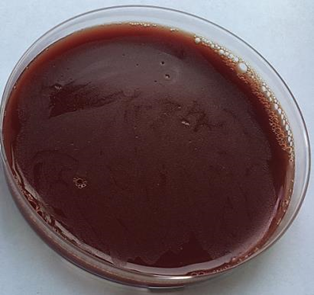


**B**


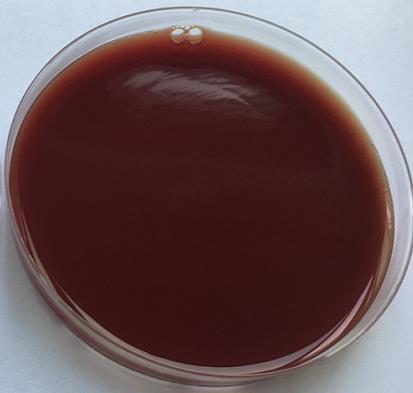

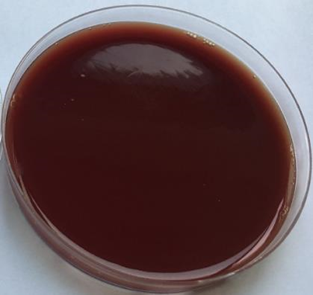


**C**


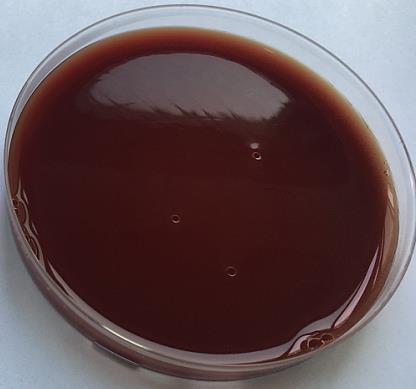

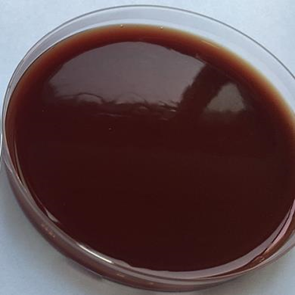


**D**

**
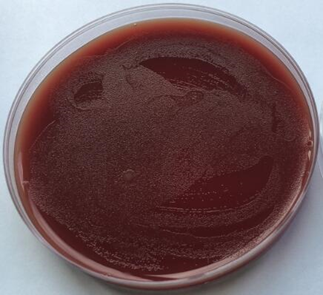

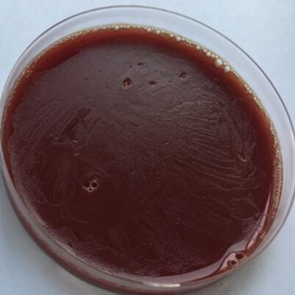
**

**E**


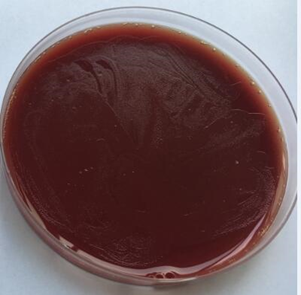

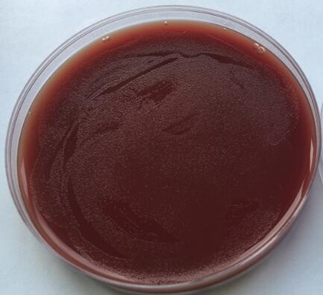


**F**

**
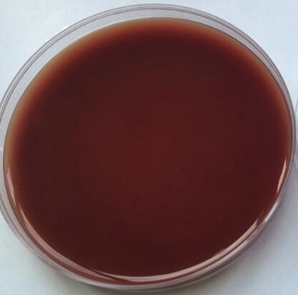

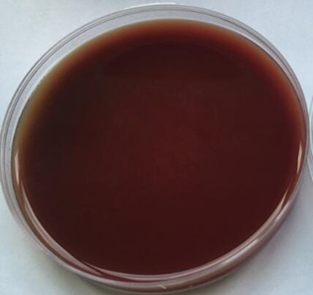
**

**G**

**
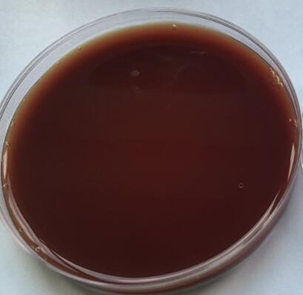

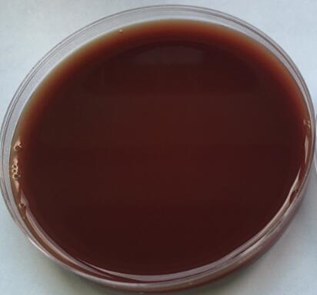
**

**H**

**Fig S2 Effects of Pal and Met on the growth of *H. pylori* NCTC 26695 under neutral condition (pH 7.4) at different concentrations by the agar dilution method.** After 72 h, the effects of Pal and Met on the growth of *H. pylori* were determined. **A**. Control group for Pal; **B**. Pal (150 µg/mL); **C**. Pal (200 µg/mL); **D**. Control group for Met; **E**. Met (1 µg/mL); **F**. Met (2 µg/mL); **G**. Met (4 µg/mL).


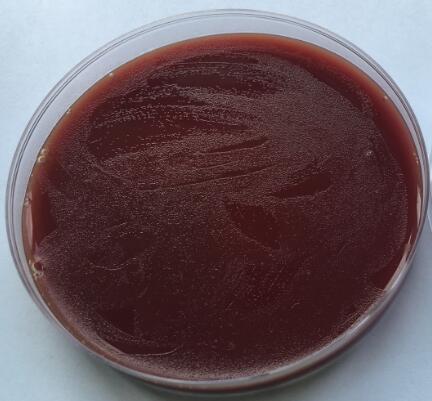

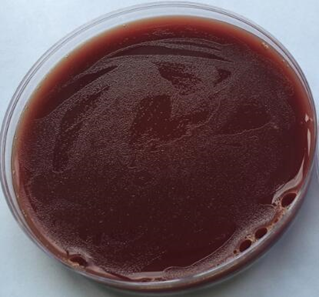


A


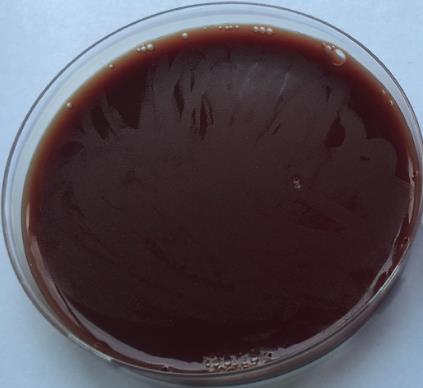

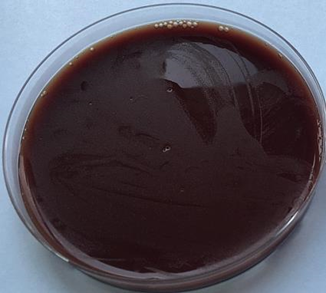


B


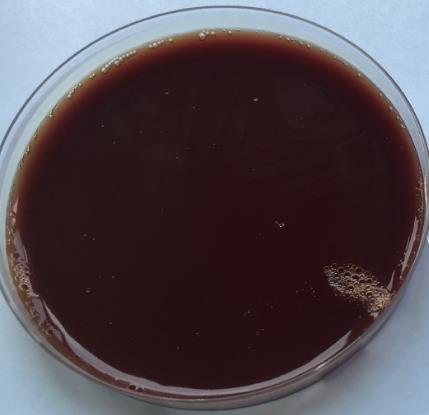

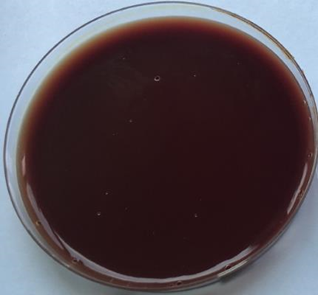


**C**

**
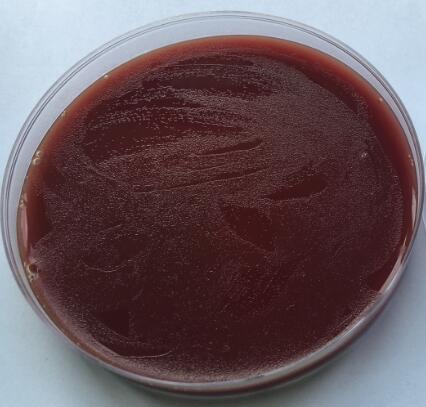

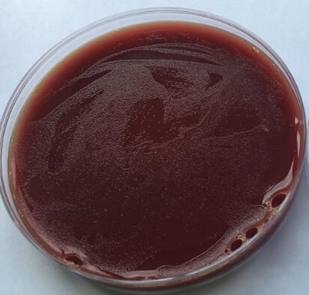
**

**D**

**
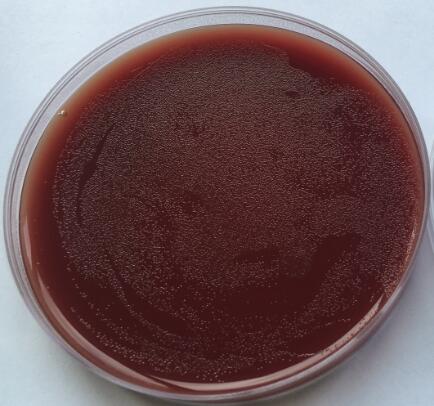

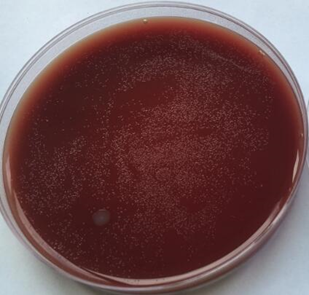
**

**E**

**
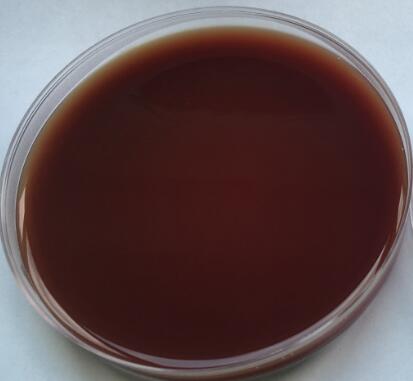

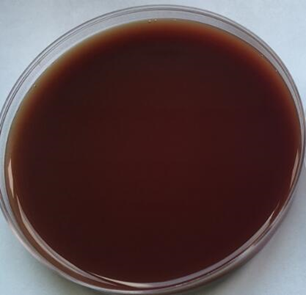
**

**F**

**
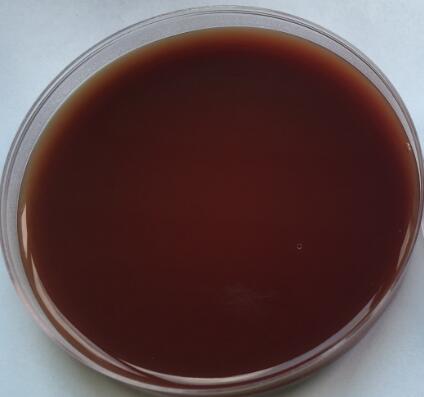

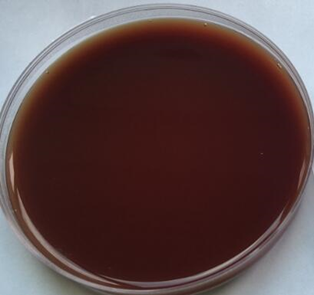
**

**G**

**Fig S3 Effects of Pal and Met on the growth of *H. pylori* SS1 under neutral condition (pH 7.4) at different concentrations by the agar dilution method.** After 72 h, the effects of Pal and Met on the growth of *H. pylori* were determined. **A**. Control group for Pal; **B**. Pal (75 µg/mL); **C**. Pal (100 µg/mL); **D**. Pal (125 µg/mL); **E**. Control group for Met; **F**. Met (0.25 µg/mL); **G**. Met (0.5 µg/mL); **H**. Met (1 µg/mL).


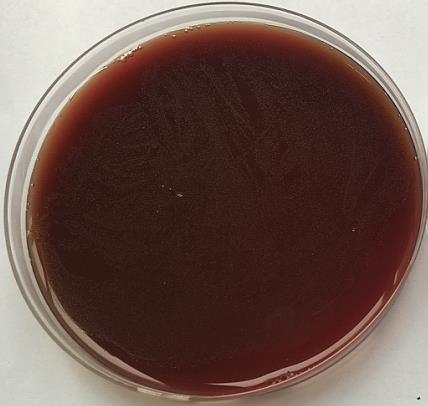

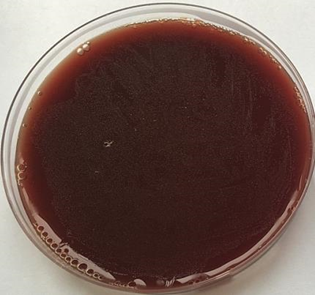


**A**


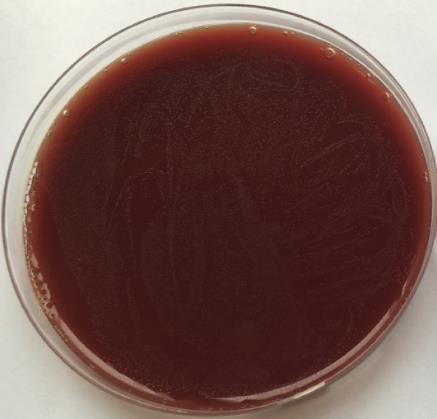

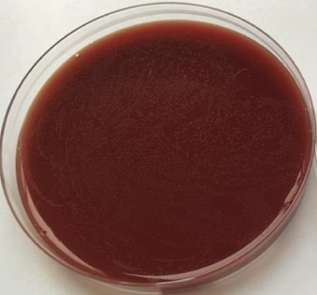


**B**


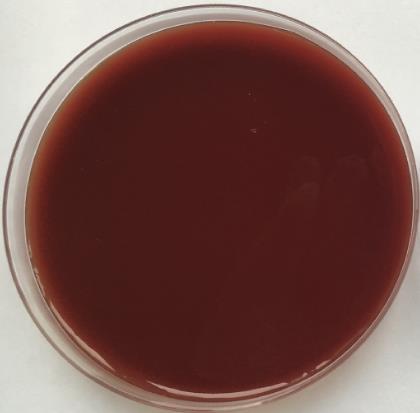

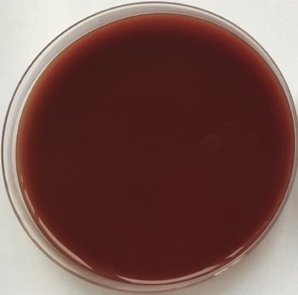


**C**

*
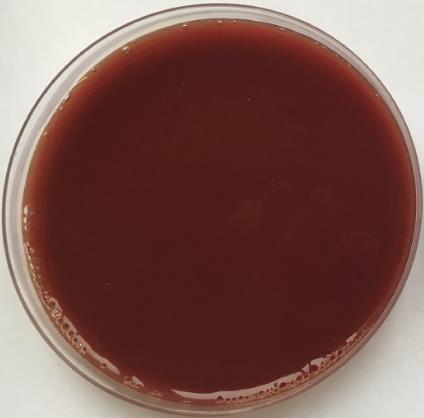

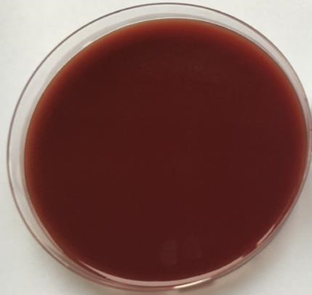
*

**D**


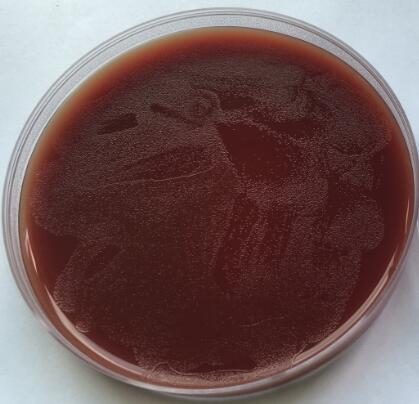

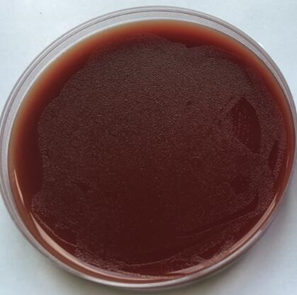


**E**


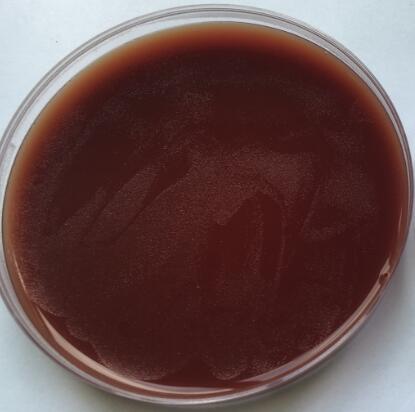

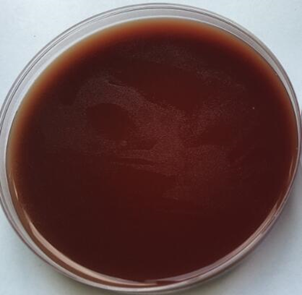


**F**


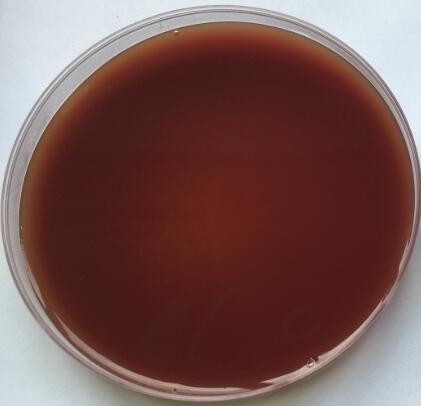

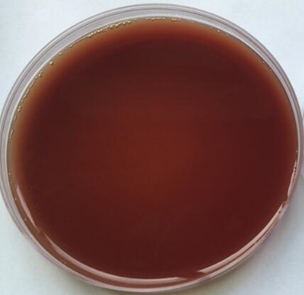


**G**


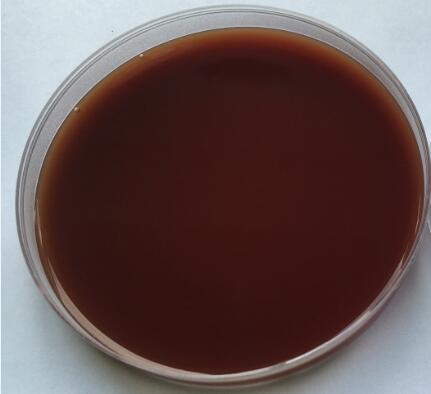

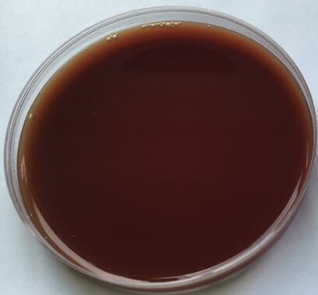


**H**

**Fig S4 Effects of Pal and Met on the growth of *H. pylori* ICDC 111001 under neutral condition (pH 7.4) at different concentrations by the agar dilution method.** After 72 h, the effects of Pal and Met on the growth of *H. pylori* were determined. **A**. Control group for Pal; **B**. Pal (75 µg/mL); **C**. Pal (100 µg/mL); **D**. Pal (125 µg/mL); **E**. Control group for Met; **F**. Met (1 µg/mL); **G**. Met (2 µg/mL); **H**. Met (4 µg/mL).


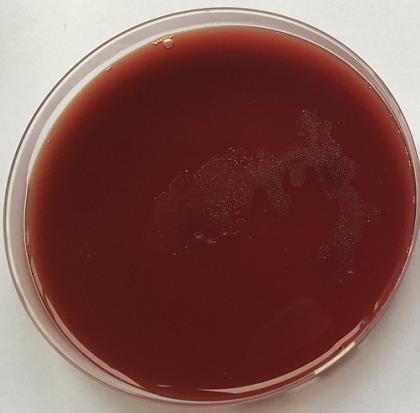

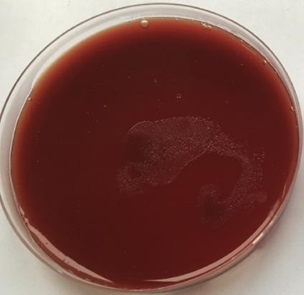


**A**


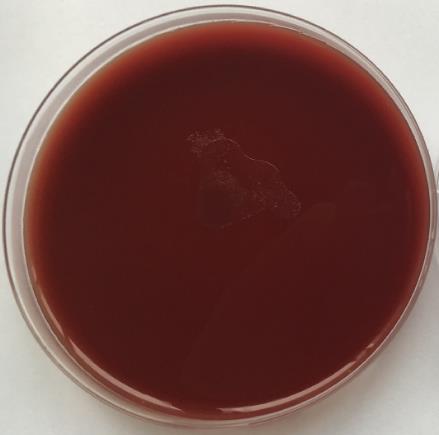

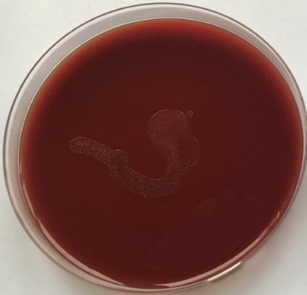


**B**


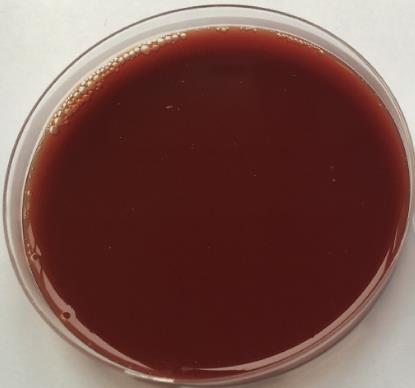

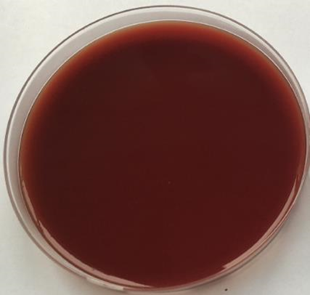


**C**


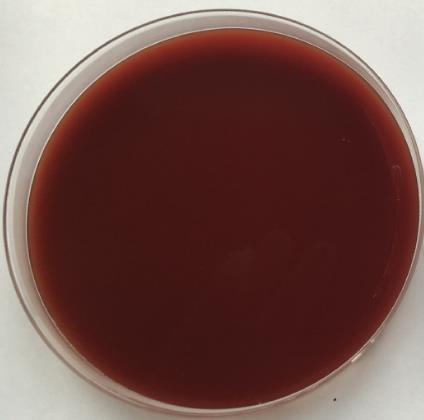

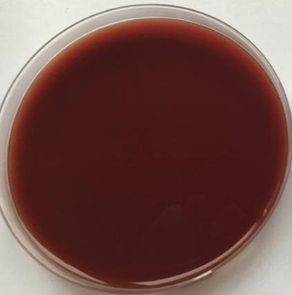


**D**


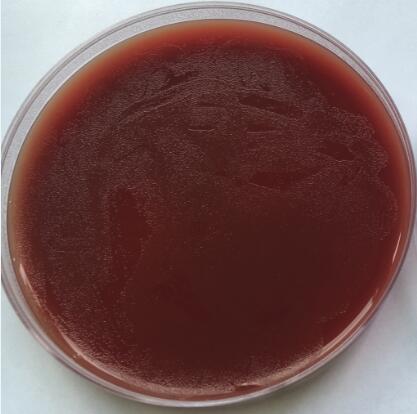

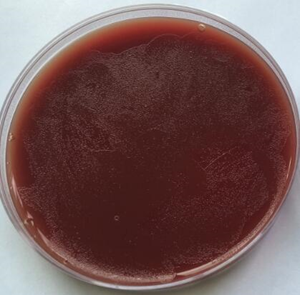


**E**

**
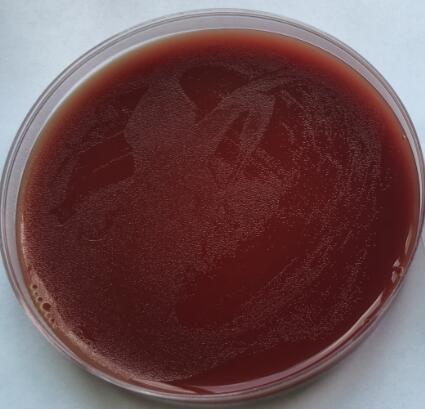

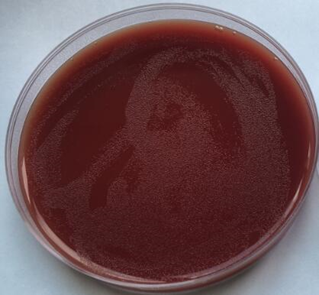
**

**F**

**
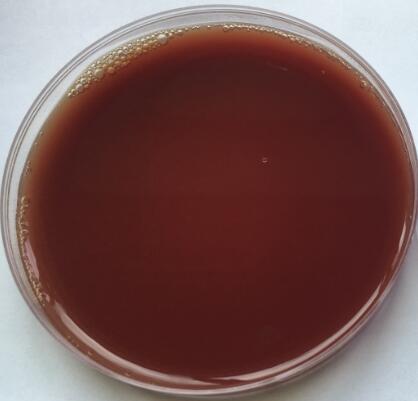

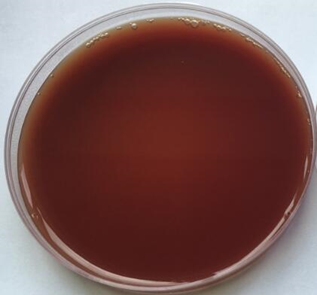
**

**G**

**
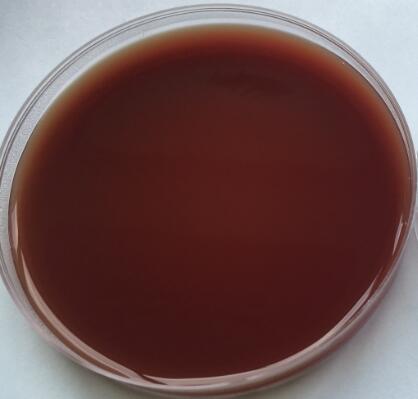

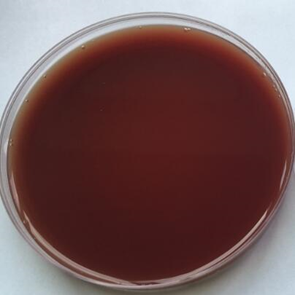
**

**H**

**Fig S5 Effects of Pal and Met on the growth of *H. pylori* ATCC 43504 under neutral condition (pH 5.3) at different concentrations by the agar dilution method.** After 72 h, the effects of Pal and Met on the growth of *H. pylori* were determined. **A**. Control group for Pal; **B**. Pal (50 µg/mL); **C**. Pal (75 µg/mL); **D**. Pal (100 µg/mL); **E**. Control group for Met; **F**. Met (0.25 µg/mL); **G**. Met (0.5 µg/mL); **H**. Met (1 µg/mL).

**
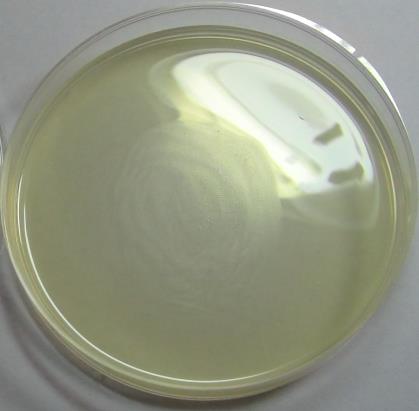

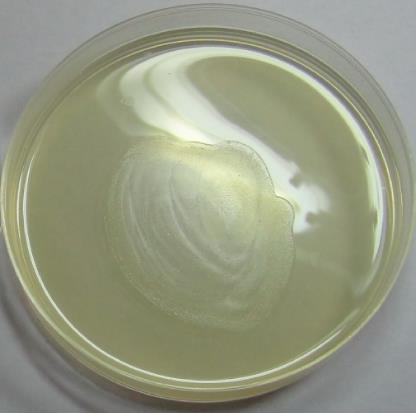
**

**A**

**
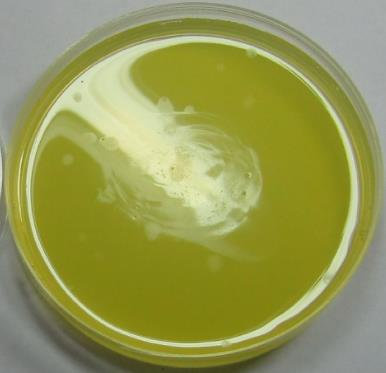

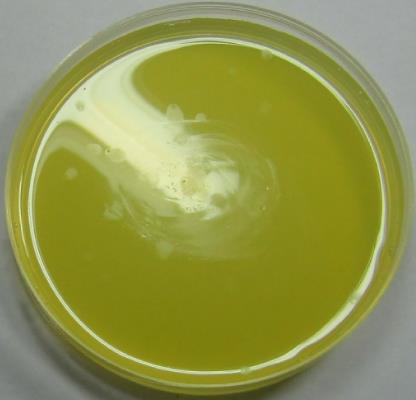
**

**B**

**
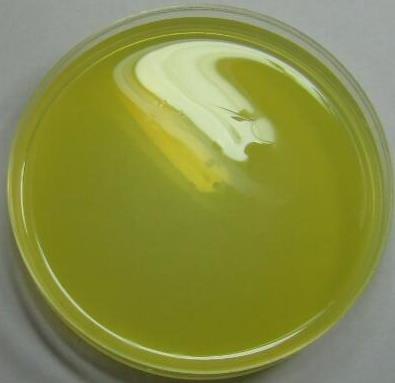

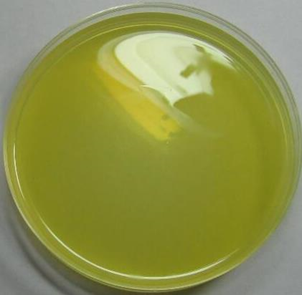
**

**C**

**
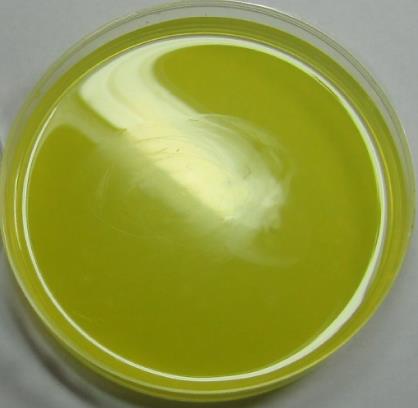

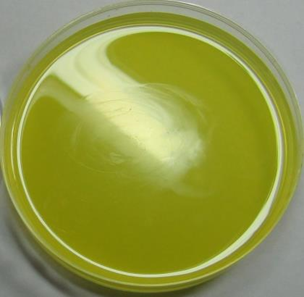
**

**D**

**
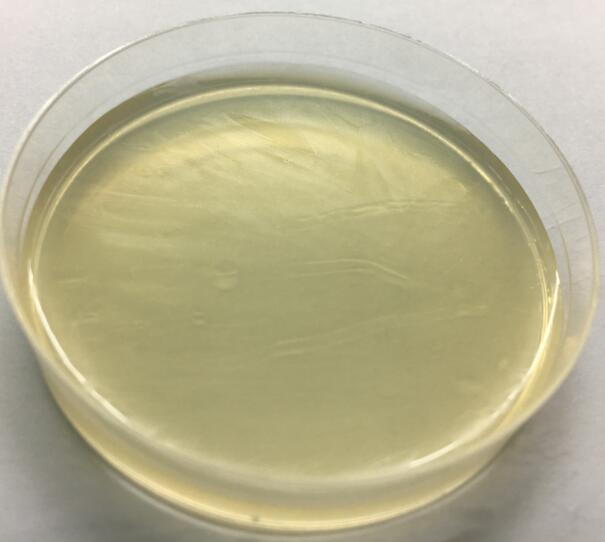

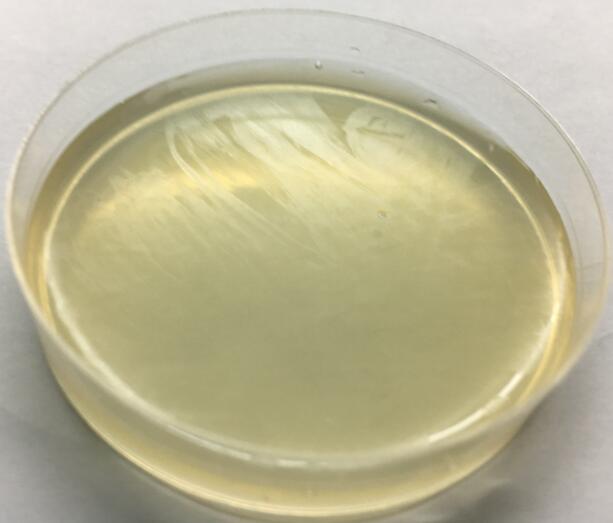
**

**E**

**
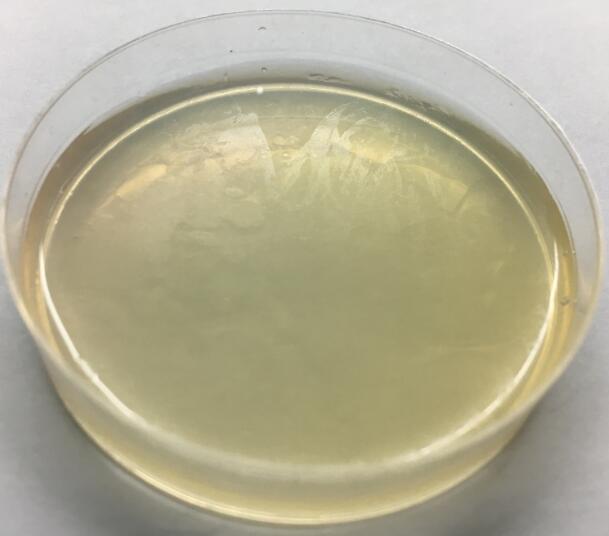

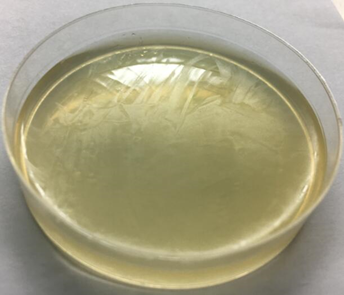
**

**F**

**
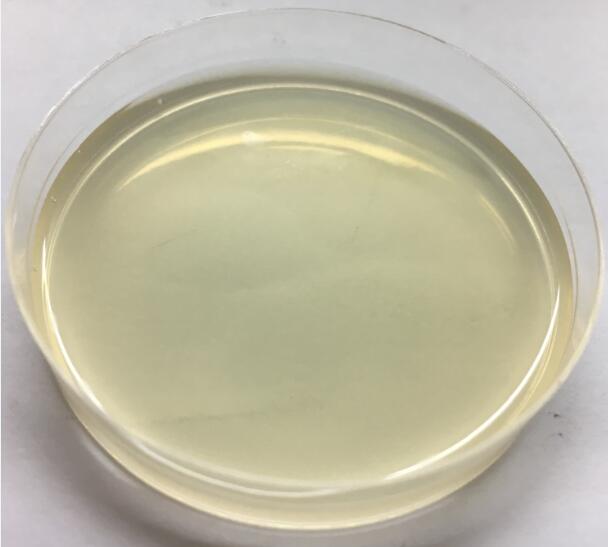

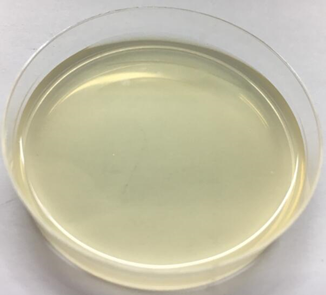
**

**G**

**
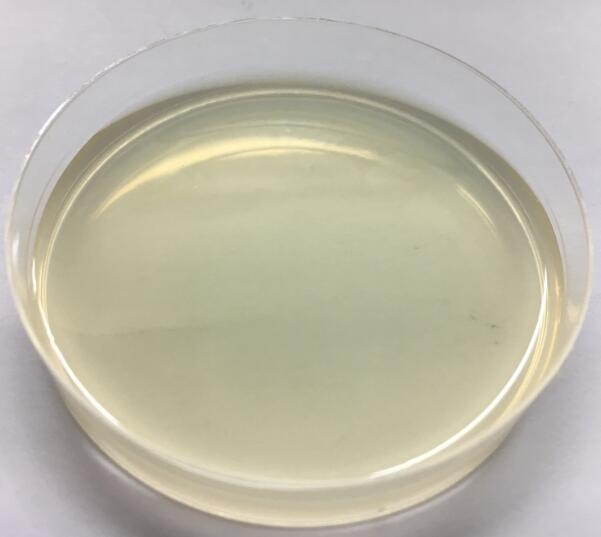

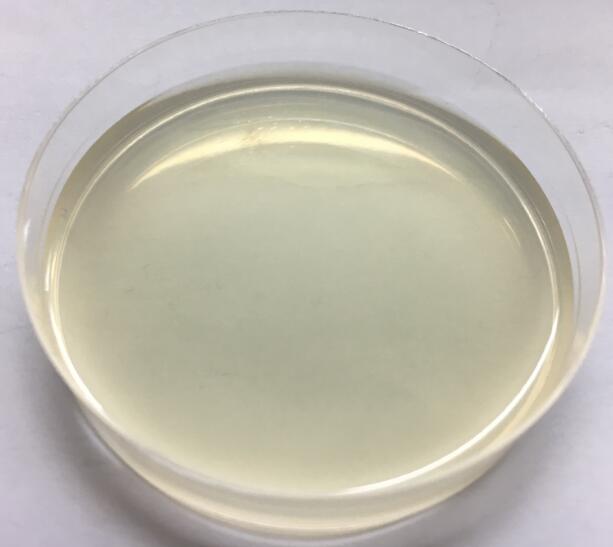
**

**H**

**Fig S6 Effects of Pal and Met on the growth of *H. pylori* NCTC 26695 under neutral condition (pH 5.3) at different concentrations by the agar dilution method.** After 72 h, the effects of Pal and Met on the growth of *H. pylori* were determined. **A**. Control group for Pal; **B**. Pal (75 µg/mL); **C**. Pal (100 µg/mL); **D**. Pal (125 µg/mL); **E**. Control group for Met; **F**. Met (1 µg/mL); **G**. Met (2 µg/mL); **H**. Met (4 µg/mL).


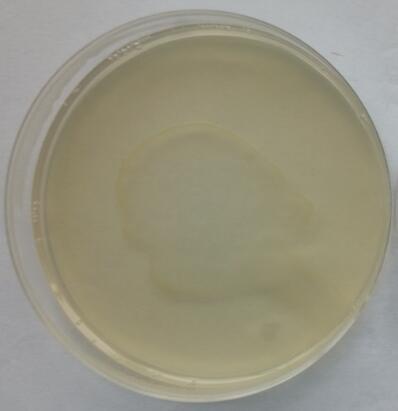

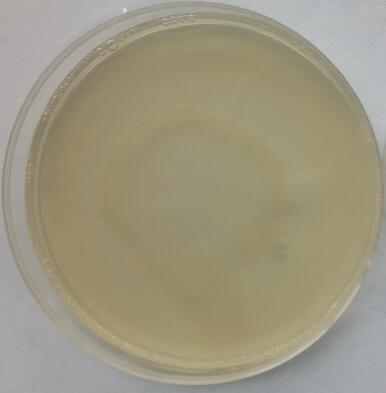


**A**


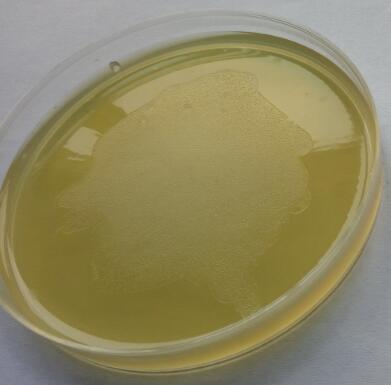

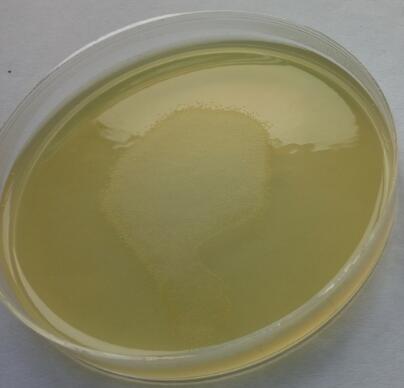


**B**


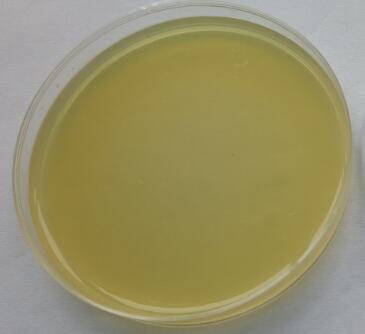

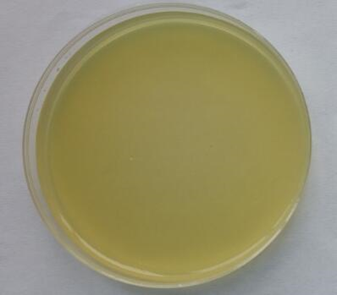


**C**


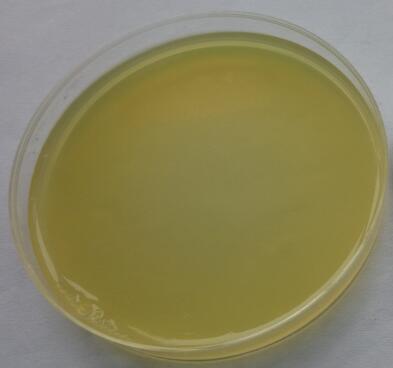

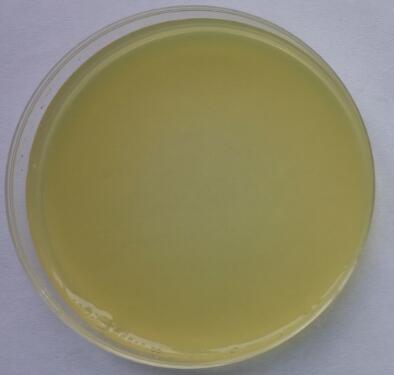


**D**


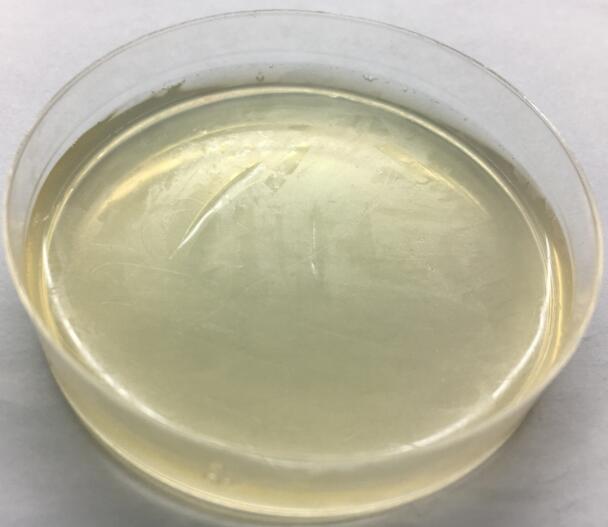

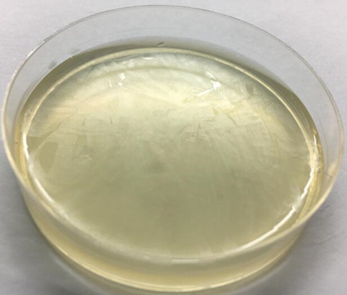


**E**


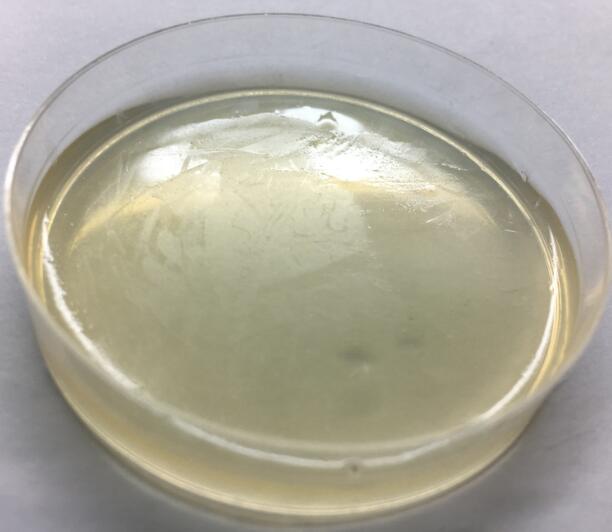

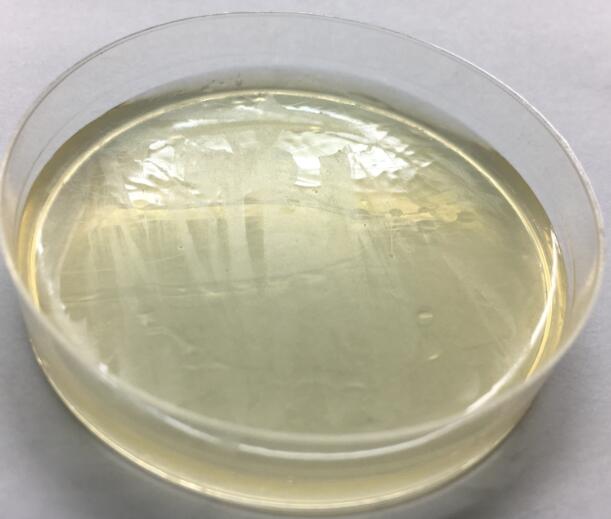


**F**


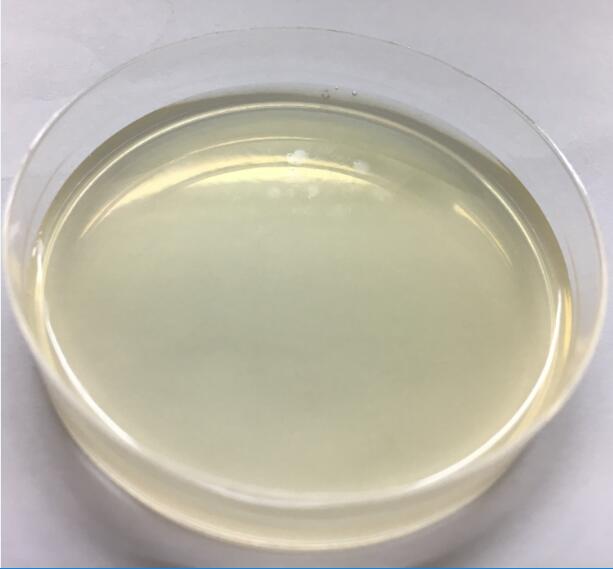

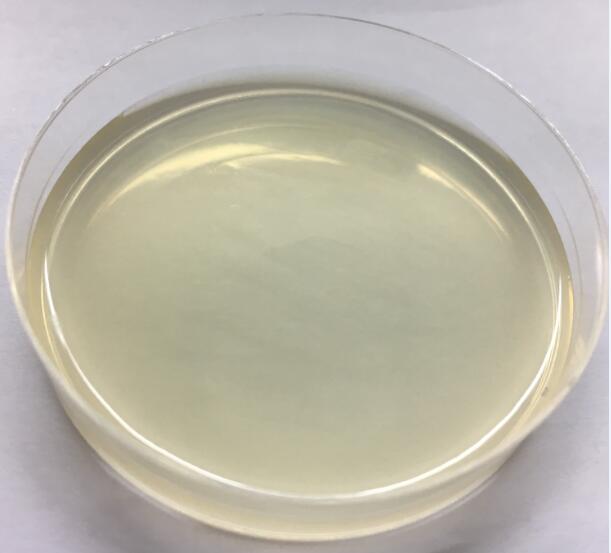


G


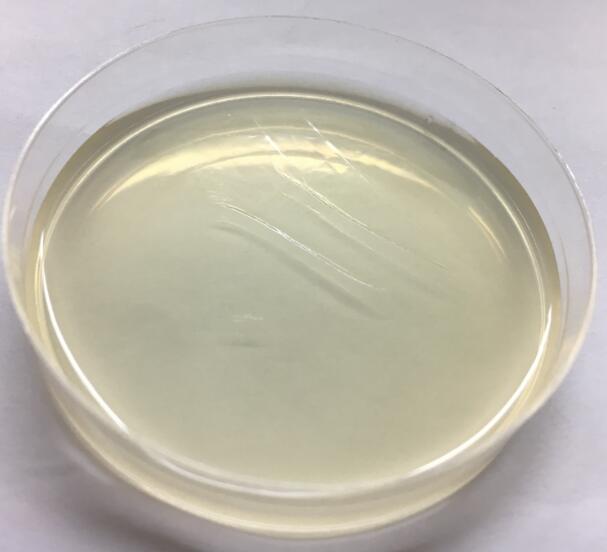

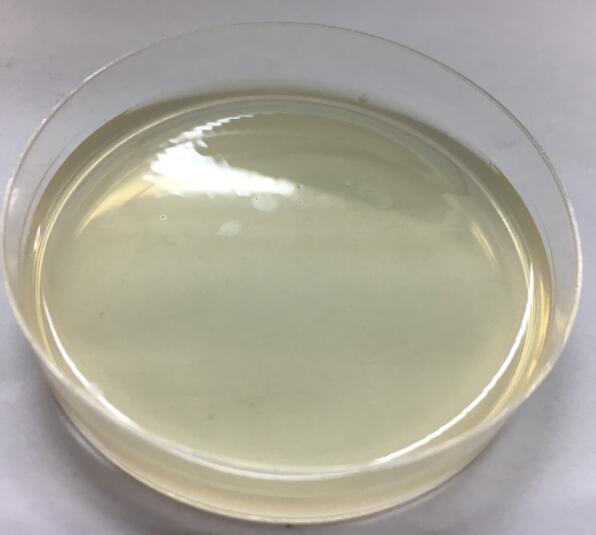


H

**Fig S7 Effects of Pal and Met on the growth of *H. pylori* SS1 under neutral condition (pH 5.3) at different concentrations by the agar dilution method.** After 72 h, the effects of Pal and Met on the growth of *H. pylori* were determined. **A**. Control group for Pal; **B**. Pal (50 µg/mL); **C**. Pal (75 µg/mL); **D**. Pal (100 µg/mL); **E**. Control group for Met; **F**. Met (0.25 µg/mL); **G**. Met (0.5 µg/mL); **H**. Met (1 µg/mL).


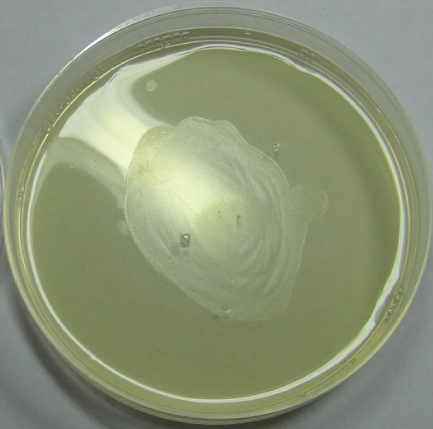

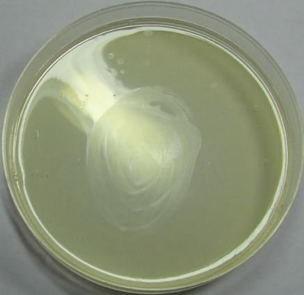


**A**


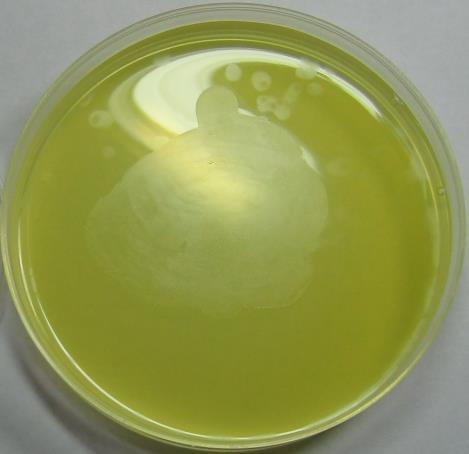

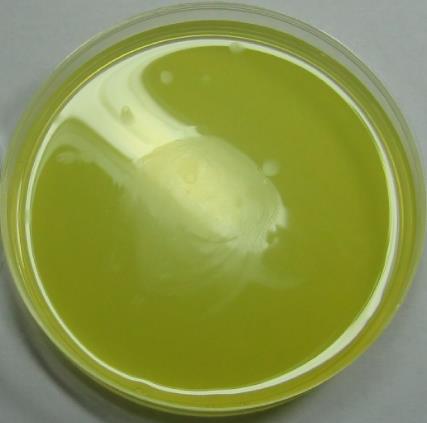


**B**


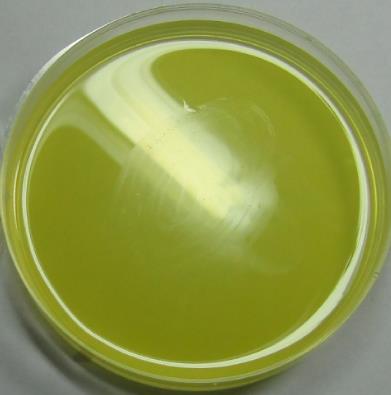

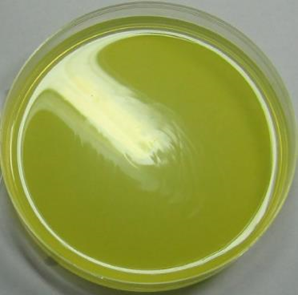


**C**

**D**

**E**

**F**

**G**

**H**

**Fig S8 Effects of Pal and Met on the growth of *H. pylori* ICDC 111001 under neutral condition (pH 5.3) at different concentrations by the agar dilution method.** After 72 h, the effects of Pal and Met on the growth of *H. pylori* were determined. **A**. Control group for Pal; **B**. Pal (50 µg/mL); **C**. Pal (75 µg/mL); **D**. Pal (100 µg/mL); **E**. Control group for Met; **F**. Met (1 µg/mL); **G**. Met (2 µg/mL); **H**. Met (4 µg/mL).

**A**

**B**

**C**

**D**

**E**

**F**

**G**

**H**
